# The Role of Advanced MRI Sequences in the Diagnosis and Follow-Up of Adult Brainstem Gliomas: A Neuroradiological Review

**DOI:** 10.3390/tomography9040122

**Published:** 2023-08-18

**Authors:** Alessia Guarnera, Andrea Romano, Giulia Moltoni, Tamara Ius, Serena Palizzi, Allegra Romano, Daniele Bagatto, Giuseppe Minniti, Alessandro Bozzao

**Affiliations:** 1 Neuroradiology Unit, NESMOS Department Sant’Andrea Hospital, La Sapienza University of Rome, Via di Grottarossa, 1035-1039, 00189 Rome, Italy; andrea.romano@uniroma1.it (A.R.); giulia.moltoni@uniroma1.it (G.M.); serenapalizzi@gmail.com (S.P.); allegraromano@libero.it (A.R.); alessandro.bozzao@uniroma1.it (A.B.); 2 Neuroradiology Unit, Imaging Department, Bambino Gesù Children’s Hospital, Piazza Sant’Onofrio 4, 00165 Rome, Italy; 3 Neurosurgery Unit, Head-Neck and NeuroScience Department, University Hospital of Udine, Piazzale Santa Maria della Misericordia 15, 33100 Udine, Italy; tamara.ius@gmail.com; 4 Neuroradiology Unit, Department of Diagnostic Imaging, University Hospital of Udine, Piazzale Santa Maria della Misericordia 15, 33100 Udine, Italy; daniele.bagatto@gmail.com; 5 Department of Radiological Sciences, Oncology and Anatomical Pathology, Division of Radiotherapy, La Sapienza University of Rome, 00161 Rome, Italy; giuseppe.minniti@uniroma1.it; 6 IRCCS Neuromed, 86077 Pozzilli, Italy

**Keywords:** brainstem glioma, 2021 WHO classification, diffuse intrinsic low-grade gliomas, enhancing malignant gliomas, localized tectal gliomas, MRI, DWI, DTI, PWI, MRS

## Abstract

The 2021 WHO (World Health Organization) classification of brain tumors incorporated the rapid advances in the molecular, genetic, and pathogenesis understanding of brain tumor pathogenesis, behavior, and treatment response. It revolutionized brain tumor classification by placing great emphasis on molecular types and completely splitting adult-type and pediatric-type diffuse gliomas. Brainstem gliomas (BSGs) are the leading primary tumors of the brainstem, although they are quite uncommon in adults compared with the pediatric population, representing less than 2% of adult gliomas. Surgery is not always the treatment of choice since resection is rarely feasible and does not improve overall survival, and biopsies are not generally performed since the location is treacherous. Therefore, MRI (Magnetic Resonance Imaging) without and with gadolinium administration represents the optimal noninvasive radiological technique to suggest brainstem gliomas diagnosis, plan a multidisciplinary treatment and for follow-up evaluations. The MRI protocol encompasses morphological sequences as well as functional and advanced sequences, such as DWI/ADC (Diffusion-Weighted Imaging/Apparent Diffusion Coefficient), DTI (Diffusion Tensor Imaging), PWI (Perfusion-Weighted Imaging), and MRS (Magnetic Resonance Spectroscopy), which improve the accuracy of the diagnosis of BSGs by adding substantial information regarding the cellularity, the infiltrative behavior toward the v fiber tracts, the vascularity, and the molecular changes. Brainstem gliomas have been divided into four categories on the basis of their MRI radiological appearance, including diffuse intrinsic low-grade gliomas, enhancing malignant gliomas, localized tectal gliomas, and other forms. The aim of our review is to provide insight into the role of advanced MRI sequences in the diagnosis and follow-up of adult brainstem gliomas.

## 1. Introduction

The 2021 WHO classification of brain tumors incorporated the rapid advances in the molecular, genetic, and pathogenesis understanding of brain tumor pathogenesis, behavior, and treatment response. It revolutionized brain tumor classification by placing great emphasis on molecular types and completely splitting adult-type and pediatric-type diffuse gliomas [1,2,3].

Although they are rare in adults compared with children (less than 2% of adult gliomas), brainstem gliomas (BSG) are the most prevalent primary tumors of the brainstem [4].

The optimal treatment is radiotherapy, which may be associated with or followed by chemotherapy. Surgery is seldom performed since it is an invasive therapy that does not pair with extended overall survival. Similarly, the peculiar location of the tumors may limit biopsy acquisition [4,5,6]. The EANO 2021 guidelines on the diagnosis and treatment of diffuse gliomas in adulthood identified MRI without and with gadolinium-based contrast agents as the first choice for diagnostic imaging [1]. The optimal MRI protocol includes both morphological and advanced sequences to diagnose brainstem gliomas, suggests the subtype of the tumor, plans the best treatment for the patient, and identifies early recurrence.

Our goal in this review is to describe the role of advanced MRI sequences in the diagnosis and follow-up of the main categories of adult brainstem gliomas: diffuse intrinsic low-grade gliomas (DILGGs), enhancing malignant gliomas (EMGs), focal tectal gliomas (FTGs), and other types [4,5,6,7].

## 2. MRI Protocol

An optimal and comprehensive MRI protocol, including advanced MRI sequences, is key for the radiologist to identify the brainstem glioma subtype, suggest tumor progression, and guide tumor biopsy and surgical treatment. Finally, MRI is pivotal in the follow-up since it may distinguish between recurrent disease and post-treatment changes.

A comprehensive MRI protocol should encompass morphological and advanced MRI sequences.

The MRI protocol we perform at our institution on our 3T MRI scanner (Achieva, Philips Medical System, Best, The Netherlands) with a 32-channel brain coil (L, W, H/length, width, height: 440 mm × 330 mm × 370 mm) includes axial TSE/turbo spin-echo T2 (TR/repetition time 3000 ms, TE/echo time 89 ms, ST/slice thickness 4 mm); sagittal 3D FLAIR/Fluid-Attenuated Inversion Recovery (TR 4600 ms, TE 328 ms, ST 1 mm—isotropic voxel); axial T1 IR/inversion recovery (TR 2000 ms, TE 20 ms, TI/inversion time 50 ms, ST 4 mm); axial DWI/Diffusion-Weighted Imaging (TR 3150 ms, TE 47 ms, FA/flip angle 75°, ST 4 mm, b0-500-1000 s/mm^2^); 64-direction axial DTI/Diffusion Tensor Imaging (TR 8869 ms, TE 73 ms, ST 2.10 mm); and sagittal precontrast 3D T1 IR (TR 8.2 ms, TE 3.8 ms, ST 1 mm—isotropic voxel). After contrast administration, the following sequences are acquired: sagittal postcontrast 3D T1 IR (TR 8.2 ms, TE 3.8 ms, ST 1 mm—isotropic voxel); coronal T1 SPIR/Spectral Presaturation with Inversion Recovery (TR 25 ms, TE 2.4 ms, ST 1 mm—isotropic voxel); axial T1 SE/spin-echo (TR 8600 ms, TE 10 ms, ST 4 mm); and DSC/dynamic susceptibility contrast PWI/Perfusion WI (TR 1710 ms, TE 40 ms, ST 4 mm). MRS encompasses SV/single-voxel PRESS/Point Resolved Spectroscopy (TR 2000 ms, TE 144 ms, voxel 20 × 20 × 20) and 2D PRESS (TR 2000 ms, TE 144 ms, voxel 10 × 10 × 10).

Morphological sequences are almost universally performed, as they require a limited amount of time and do not require advanced and expensive post-processing software. Usually, 3D T1 MPRAGE is superior to SE T1WI since it may easily allow a post-acquisition reconstruction on three planes and a high-quality detailed definition. On the other hand, the sequence requires a higher time of acquisition. T2 and FLAIR WI are considered standard acquisitions for the optimal definition of the tumor and surrounding structure signals. The identification of blood, which may be found after tumor biopsy or after treatment and in high-grade subtypes, such as EMGs, is facilitated by the further acquisition of an SWI (susceptibility-weighted image) or a GRE T2* (gradient echo). The sensitivity and specificity of an SWI are superior to those of a GRE T2*, yet SWIs are not supported by all the scanners. Advanced sequences are key to a better understanding of the tumor subtype, response to therapy, and recurrence. Since the abovementioned sequences are time-consuming, demand patient compliance, and require expensive acquisition and post-processing software, they are not performed at all institutions and are frequently limited to high-risk patients. We report the DWI/ADC among the most performed and almost standard MRI advanced sequences. DWI/ADC provides crucial information on tumor cellularity and may guide the differential diagnosis between high- and low-grade tumors. A twin sequence is DTI, often performed to distinguish between infiltrated and displaced white matter fibers. The distinction is essential for performing biopsies and planning surgery since displaced white matter fibers are more prone to indicate a low-grade tumor and should be preserved from excision. On the other hand, infiltrated fibers relate to an invasive, high-grade tumor and do not require surgical preservation. Therefore, DTI sequence may be acquired before tumor treatment to plan the best therapy for patients. To distinguish between low- and high-grade tumors, we may also perform the PWI sequence, which requires the administration of a contrast agent like gadolinium and distinguishes between well-perfused and low-perfused tumors. Moreover, PWI represents an optimal sequence to identify early recurrence compared to post-treatment changes. Therefore, PWI may be of use both in the diagnostic phase and in the follow-up phase. It is commonly performed in association with a post-contrast T1WI, which identifies focal areas of contrast-enhancement. PWI is superior to post-contrast T1W1 since contrast enhancements may refer to brain–blood barrier alterations, post-treatment changes, or recurrence. As abovementioned, PWI reflects perfusion and guides the differential diagnosis. Finally, MRS is an advanced sequence which carries molecular information on the tumor and the surrounding tissues. As the 2021 WHO classification highlighted, molecular alterations are crucial and may precede tumor radiological appearance and clinical symptoms. Tumor early identification and treatment are crucial to extending patients’ overall survival [1,2,3,4,5,6,7].

## 3. Brainstem Gliomas

Apart from their peculiar radiological presentation, BSGs are characterized by different epidemiology, symptoms and prognosis, epicenter and spreading, and anatomopathological features (Table 1).

### 3.1. Diffuse Intrinsic Low-Grade Gliomas (DILGGs)

**Figure 1:**
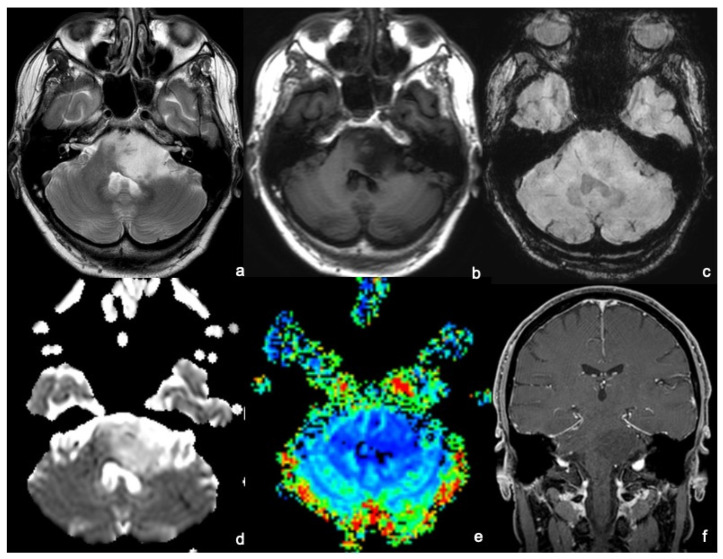
An MRI of a 45-year-old man presenting with a long-lasting headache and dizziness related to a diffuse intrinsic low-grade glioma. MRI shows a bulky lesion of the left pons extending: anteriorly, partially obliterating the prepontine cistern and contacting the patent basilar; posteriorly to invade the left–middle cerebellar peduncle and to compress the IV ventricle; and medially to the right hemipons. The lesion appears to be inhomogeneously hyperintense on T2WI (**a**) and hypointense on T1WI (**b**), without hemorrhagic foci on SWI (**c**), and without diffusion restriction foci on the ADC map (**d**). After gadolinium administration, the tumor does not enhance on T1WI (**f**), and there is no increased perfusion on the rCBV map (**e**). The anatomopathological analysis demonstrated that the lesion was a grade 2—2021 WHO classification of brain tumors.

DILGGs appear as infiltrative, ill-margined tumors causing the enlargement of the brainstem and obliteration of adjacent cisterns, with typical spreading along the cerebellar peduncles to the cerebellar hemispheres posteriorly, to the midbrain and thalamus cranially, and to the medulla caudally. Rarely pontine-centered tumors display an exophytic growth and encase the basilar artery anteriorly. Morphological sequences poorly define DILGGs’ features since they only show a T1WI hypointense and T2WI hyperintense lesion with no enhancement after contrast administration [4,5,6,7] (Figure 1a–c,f).

DWI/ADC has been among the first functional sequences associated with tumor grade and shows a correlation between ADC values and tumor cellularity. DILGGs appear as hypocellular tumors associated with peripheral edema, characterized by no diffusion restriction on DWI and high signal on ADC maps (Figure 1d). These findings match DTI features, identifying a low FA (fractional anisotropy). DWI and DTI may detect early tumor progression from low to high grade by identifying focal areas of diffusion restriction, ADC hypointensity, and higher values of FA. Despite continuous technical advancement in neuroimaging and operative techniques, the role of surgery for BSGs is still controversial and slightly documented. BSG surgery is challenging due to BSG intricate vascular and functional anatomy. Therefore, DTI fiber tracking has proven to be a crucial tool for surgical biopsy and planning neurosurgery since it allows the noninvasive visualization of the brain’s anatomical structures and guides the procedures by choosing the best path with minimal risks. Color-coded images, superimposed to morphological images (T1WI or T2WI), help to visualize the multiple WM tracts passing through the brainstem. Commonly, DILGGs cause a displacement of WM tracts, while evidence of fiber disruption suggests tumor progression [8,9,10,11,12].

Tumor grading is also associated with tumor neovascularity, which optimally better reflects tumor prognosis. Unfortunately, brainstem location in the posterior fossa close to the skull base and the presence of multiple perforator arteries as main vessels may impair PWI studies, whose most used parameters are rCBV (relative cerebral blood volume) and rCBF (relative cerebral blood flow) (Figure 1e). The crucial role of PWI is in identifying the focal foci of increased neovascularity, appearing as areas of increased rCBV and reflecting the anaplastic transformation of the low-grade tumor. In DILGGs, perfusion parameters are similar to adjacent WM. These parameters are also pivotal radiological features to investigate post-treatment response by differentiating post-radiation changes and tumor recurrence [5,8,13,14].

Single-voxel, and more recently, multivoxel MRS, has improved the accuracy in DILGGs’ evaluation of tumors >20 mm. On the other hand, we report that the sequence sensitivity and specificity may be impaired by the small dimensions of the anatomical structures and the proximity to posterior fossa bony structures. DILGGs typically show a low NAA (N-acetyl aspartate) and a high Cho (choline) peak. MRS helps in identifying the subtle changes in tumor progression by showing a progressive lowering of the NAA peak, increasing in the Cho peak, and evidence of lactate and lipid peaks [14,15,16].

The optimal treatment is represented by radiotherapy (RT), leading to a significant clinical response, which does not pair with a detectable imaging improvement occurring only in 19% of cases [4]. RT is often associated with glucocorticoids, which mitigate edema-related symptoms [17].

Surgery has a limited role in DILGGs therapy since total tumor resection is impossible due to the extensive spread and partial resection carrying high morbidity. When patients present with the typical diffuse, infiltrating, and non-enhancing DILGGs, biopsy is not required, and MRI is the diagnostic option of choice [5,18].

Chemotherapy’s role remains uncertain. In particular, temozolomide (TMZ) is limited to salvage therapy with moderate effectiveness since DILGGs are low-grade tumors. Bevacizumab showed a discrete clinical and radiological improvement in recurrent disease after TMZ and RT failure [19,20,21].

Post-treatment changes are not always easy to distinguish from recurrent and progressive disease and anaplastic transformation, although advanced MRI sequences may help to reach the correct diagnosis. RT induces necrotic and cystic changes of the tumor, which may be improperly associated with a progressive disease. Contrast-enhanced T1 is insufficient to distinguish between tumor recurrence and RT transformation. However, PWI does not show a perfusion increase since contrast enhancement is related to brain–blood barrier disruption. Treatment may also induce intra-tumoral hemorrhage, easily identified by GRE T2* (gradient-echo) or SWI (susceptibility WI) sequences [9,13]. DWI/ADC, DTI, PWI, and MRS are pivotal to the early identification of anaplastic transformation [5,14]. Tumor progression encompasses the infiltration of white matter fibers, identified at DTI and tractography, and the progressive enlargement of the tumor, showing contrast-enhancing areas and intra-tumoral necrosis. In advanced stages, leptomeningeal dissemination is appreciable in 13–15% of patients. DWI/ADC sequence shows restricted diffusion caused by the increased tumor cellularity and pairs with the increase in neovascularization at PWI [7,22]. Multivoxel MRS may help to identify RT response and relapse. In RT response, Cho/NAA peak decreases, and there is a loss of lactate and lipid peaks, while in the case of disease relapse, Cho/NAA peak increases, and lactate and lipid peaks remain stable or increase [23].

### 3.2. Enhancing Malignant Gliomas (EMGs)

**Figure 2:**
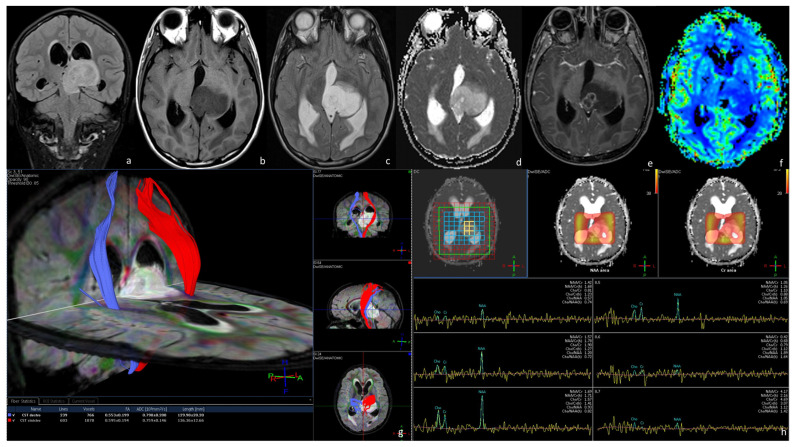
MRI of a 26-year-old man presenting with headache and nausea caused by an enhancing malignant glioma. MRI shows a left mesencephalic-centered lesion extending to the left thalamus, appearing inhomogeneously hypointense on T1WI (**b**) and hyperintense on T2WI (**c**), and FLAIR (**a**) sequences. The lesion, surrounded by subtle edema and causing compression on the posterior part of the III ventricle and on the aqueducts of Silvius, induced an obstructive hydrocephalus with distension of the lateral ventricle and the anterior part of the III ventricle, surrounded by linear T2/FLAIR hyperintensity of the subependymal tissue, indicating cerebrospinal fluid pressure resorption. After gadolinium administration, the lesion presents a focal ring-like enhancement in the medial section surrounding necrosis (**e**). The ring-like enhancement corresponds to an increased perfusion in the rCBV map (**f**) and subtle diffusion restriction in the ADC map (**d**). The necrotic component corresponds to a decreased signal perfusion in the rCBV map (**f**) and an increased diffusivity in the ADC map (**d**). On DTI fiber tracking (**g**), we reconstructed the corticospinal tracts. The left corticospinal tract (red in (**g**)) is anteriorly dislocated compared to the healthy contralateral corticospinal tract (blue in (**g**)). Multivoxel MRS (**h**) showed a global reduction in all the peaks of principal metabolites in different regions of the lesion. The anatomopathological analysis demonstrated that the lesion was a grade 4—2021 WHO classification of brain tumors.

Malignant BSGs appear unevenly hypointense on T1WI and hyperintense on T2WI/FLAIR, relating to necrotic and hemorrhagic foci (Figure 2a–c). In 100% of cases, after contrast administration, the tumor shows intense and inhomogeneous or ring-like enhancement due to the necrotic components (Figure 2e). Diffusion restriction on DWI corresponds to hypointensity on ADC maps, suggesting high cellularity, and pairs with an increased FA on DTI (Figure 2d,g). FA values are generally altered proximally and distally to the tumor, indicating an axonal degeneration of motor and sensory pathways [11]. DTI fiber-tracking guides the identification and quantification of tumor involvement in sensory, motor, and transverse brain tracts since it shows the disruption and amputation of WM fibers, rapidly infiltrated by the tumor, and pairs with the clinical symptoms. EMGs are characterized by significant neovascularity, reflected in the increase in rCBV on PWI with cut-off values for malignancy above 2.9 (Figure 2f). MRS shows a markedly reduced NAA peak, reflecting the decreased neuronal density, and an increased Cho peak, related to the neoplastic tissue proliferation, and high peaks of lactate and lipids, indicating intra-tumoral foci of necrosis [5,8,13,14] (Figure 2h).

Since preoperative imaging is not always specific, MRI-guided brain biopsy is recommended to reach a proper diagnosis and set the optimal treatment. DTI and tractography are crucial for identifying involved white matter tracts, guiding the best pathway for brain biopsy and planning surgery. Tumors displacing WM fibers show an improved neurologic function after chemotherapy and surgery, characterized by an improvement of FA on DTI and reduced T2/FLAIR hyperintensity. WM tracts infiltration and disruption are usually followed by Wallerian degeneration, characterized by atrophy and T2/FLAIR hyperintensity of the white matter tracts and predictive of persisting neurological dysfunctions [4,24] (Figure 2g).

EMGs are usually resistant to RT since a radiological and clinical response is appreciable in only 13% of patients. The combination of TMZ and bevacizumab has shown a discrete efficacy in RT failures [19,20,21]. As already described in the treatment of DILGGs, radiation necrosis and pseudoprogression may be distinguished from tumor progression via advanced MRI sequences [5,9].

### 3.3. Focal Tectal Gliomas (FTGs)

**Figure 3:**
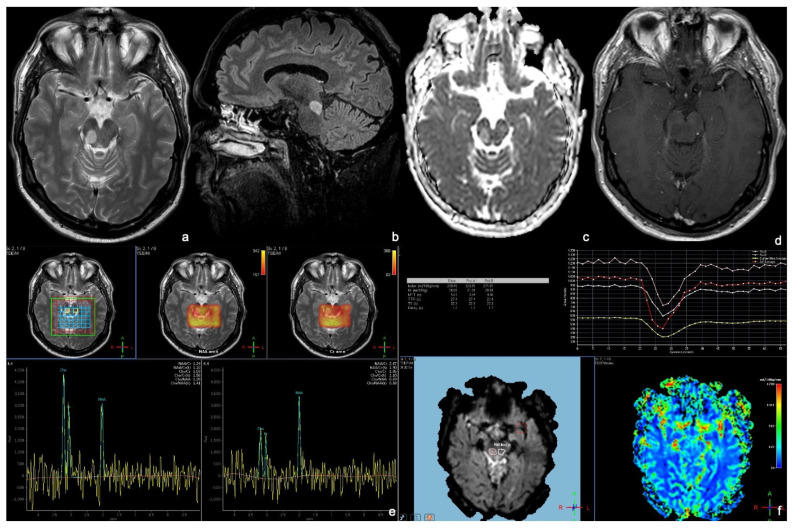
MRI of a 52-year-old nearly asymptomatic man affected by a tectal glioma and undergoing a cerebral MRI for episodic headache. MRI shows a T2 (**a**) FLAIR, (**b**) hyperintense focal lesion of the right midbrain, showing no diffusion restriction on the ADC map (**c**). After contrast administration, the lesion shows no enhancement (**d**), and there is no increase in perfusion on the rCBV map (**f**). Multivoxel MRS (**e**), performed by inserting a voxel in the center of the lesion and the other in the left midbrain, shows a low NAA peak, a high Cho peak, an elevated Cho/Cr (creatine) ratio, and a decreased NAA/Cr ratio. The anatomopathological analysis demonstrated that the lesion was of grade 1—2021 WHO classification of brain tumors.

On morphological sequences, FTGs present as focal T1 isointense and T2/FLAIR hyperintense lesions with no enhancement after contrast administration and without an increased perfusion (Figure 3a,b,d,f). No diffusion restriction on DWI/ADC is generally appreciable (Figure 3c). Notably, FTGs are often too small for performing PWI and MRS [4,5,7,9] (Figure 3e,f).

Symptomatic treatment often requires ventriculoperitoneal shunt placement or third ventriculostomy for treating the hydrocephalus. The latter is the preferred treatment as it reduces mortality and morbidity and allows a minimally invasive biopsy. Neuroendoscopic technique may be either used to biopsy the tumor or partially/fully resect the lesion. Since most patients are clinically and radiologically stable up to over ten years after ventriculoperitoneal cerebrospinal fluid shunt, there are currently two different surgical approaches. Some surgeons suggest a watchful waiting approach until a symptomatic progression, while others rely on tumor resection. In particular, contrast enhancement and tumor size are predictors of surgical intervention. A non-invasive therapy showing significant local tumor control is RT. After RT treatment, non-enhancing focal tectal gliomas may show an enhancement, which does not indicate tumor progression, yet is more likely to be associated with the brain–blood barrier disruption [4,6,25]. As mentioned above, advanced MRI sequences may guide the differential diagnosis [5,9].

### 3.4. Other Subtypes

#### 3.4.1. Exophytic Brainstem Gliomas (EBSGs)

**Figure 4:**
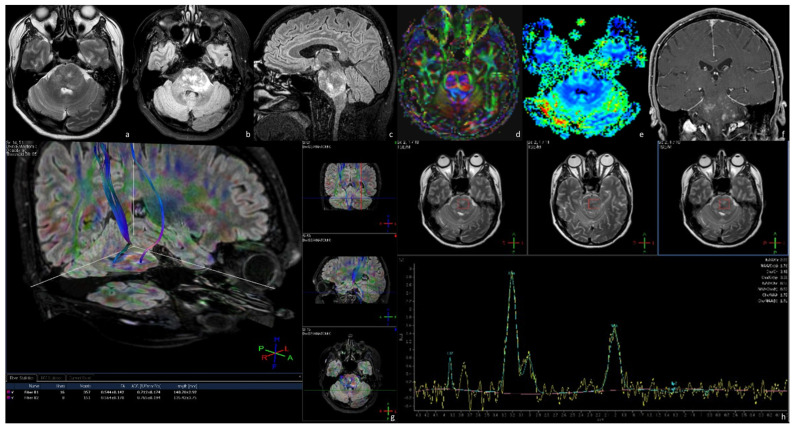
MRI of a 45-year-old man affected by an exophytic brainstem glioma presenting with a long-lasting headache and vomiting. MRI shows a bulky tumor pons extending anteriorly, partially obliterating the prepontine cistern and contacting the patent basilar artery, and posteriorly to compress the IV ventricle. The lesion appears to be inhomogeneously hyperintense on T2WI (**a**) and FLAIR (**b**,**c**). After gadolinium administration, the tumor shows an inhomogeneous enhancement on T1WI (**e**), yet there is no increase in perfusion on the rCBV map (**f**). On DTI fiber tracking (**d**,**g**), we reconstruct the corticospinal tracts. The left corticospinal tract seems to be markedly thinned compared to the contralateral one (**g**). Single-voxel MRS (**h**) acquired by positioning the voxel in the center of the lesion mainly shows an elevated peak in the choline region. The anatomopathological analysis demonstrated that the lesion was a grade 1—2021 WHO classification of brain tumors.

EBSGs present as exophytic T1WI hypointense and T2WI/FLAIR hyperintense lesions, often showing intense and homogeneous enhancement of the solid components after contrast administration (Figure 4a–c,f). These lesions sometimes present cystic components and are characterized by a slow spreading with a typical exophytic growth, which may cause a compression of the brainstem and obstruction of the cisterns. Similarly to DILGGs, advanced MRI sequences may guide the differential diagnosis between low-grade and high-grade tumors (Figure 4d,e,g,h) [4,5,7,9,13].

ESBGs are often prone to stereotactic biopsy and surgery. Subtotal tumor resection is performed to preserve neurological functions is the optimal treatment. DTI and tractography are crucial to differentiate between displaced and disrupted white matter tracts, consequently influencing surgical planning and reducing morbidity. Fiber disruption requires extensive surgery, while fiber displacement leads to tissue and neurological function preservation. Partial resection may as well be sufficient to control patients’ symptoms. Ventriculoperitoneal shunt is required to mitigate the associated hydrocephalus [4,5,9,26].

#### 3.4.2. Brainstem Gliomas Associated with Neurofibromatosis Type 1 (NFBSGs)

NFBSGs cause the enlargement of the BS, show diffuse hyperintensity on T2/FLAIR and hypointensity on T1, and may enhance after contrast administration. Tumors showing focal contrast enhancement have a higher risk of progression. MRS shows preserved neuronal marker peaks reflecting the preservation of BS neuronal elements [4,5,27,28].

Most NFBSG are asymptomatic and tend to remain stable. In case of tumor progression, more frequent in contrast-enhancing tumors, surgery or the combination of carboplatin and vincristine in surgically inaccessible tumors may represent proper treatments. Chemotherapy has the role of delaying RT, which may have a discrete response, yet at the risk of occlusive vasculopathy [4,6,28].

## 4. Conclusions

Adult brainstem gliomas represent a rare and kaleidoscopic group of various entities characterized by different clinical, radiological, and prognostic features. MRI is the imaging of choice and may avoid the need of tumor biopsy in the case of typical imaging findings, especially in DILGGs. MRI advanced sequences are pivotal to identify the subtype of BSG, for the early discovery of tumor progression, and to guide tumor biopsy and surgical treatment. Moreover, MRI has a key role in the follow-up phase since it may help to distinguish between post-treatment changes and recurrent or progressive disease.

## Figures and Tables

**Table 1:** Adult Brainstem Gliomas’ main epidemiological, clinical, and histological features.

	Diffuse Intrinsic Low-Grade Gliomas	Enhancing Malignant Gliomas	Focal Tectal Gliomas	Exophytic Brainstem Gliomas	Brainstem Gliomas Associated with Neurofibromatosis Type 1
% of Brainstem gliomas	50%	30%	<5%	rare	extremely rare
Age at diagnosis	20–50 years	>40 years			
Prognosis/median survival time	6–7 years	11–12 months	good prognosis, usually stable up to >10 years	good prognosis	good prognosis, usually asymptomatic and stable
Symptoms	cranial nerve palsies, long-tract signs	rapid onset and progression of cranial nerve palsies and long-tract signs	obstructive supra-ventricular hydrocephalus	long-lasting headache and vomiting	asymptomatic. symptomatic: intracranial hypertension, seizures and vision impairment
Epicenter	medulla (60%), pons (30%)	brainstem	midbrain	subependymal tissue adjacent to the floor of the fourth ventricle	brainstem (second most common site after the optic pathway)
Behavior and typical spreading	- infiltrative and causing enlargement of the brainstem and obliteration of adjacent cisterns; - typical spreading along the cerebellar peduncles to the cerebellar hemispheres posteriorly, to the midbrain and thalamus cranially, and to the medulla caudally; - rarely, pontine-centered tumors show an exophytic growth and encase the basilar artery anteriorly	rapidly infiltrative	- commonly small, with no infiltrative behavior; - bulky types: compression of the aqueduct of Sylvius	slow-spreading with a typical exophytic growth, which may cause compression of the brainstem and obstruction of the cisterns	- infiltrative and causing enlargement of the brainstem
Main histological type	low-grade tumors, may evolve into anaplastic astrocytomas and/or glioblatomas	anaplastic astrocytomas, glioblatomas	low-grade tumors	low-grade and high-grade variants	low-grade and high-grade variants

## Data Availability

The data are available from the corresponding author, A.G., upon reasonable request.

## Abbreviations

BSG (brainstem gliomas), MRI (Magnetic Resonance Imaging), WI (Weighted Imaging) DWI/ADC (Diffusion WI/Apparent Diffusion Coefficient), DTI (Diffusion Tensor Imaging), PWI (Perfusion WI), MRS (MR Spectroscopy), FLAIR (Fluid-Attenuated Inversion Recovery), GRE T2* (gradient-echo), SWI (susceptibility WI), DILGGs (diffuse intrinsic low-grade gliomas), FA (fractional anisotropy), rCBV (relative cerebral blood volume), rCBF (relative cerebral blood flow), EMG (enhancing malignant gliomas), FTG (focal tectal gliomas), EBSG (exophytic brainstem glioma), NFBSG (Brainstem Gliomas associated to Neurofibromatosis Type 1), RT (radiotherapy), TMZ (temozolomide), WHO (World Health Organization), NAA (N-acetylaspartate), Cho (choline), Cr (creatine), TR (repetition time), TE (echo time), IR (inversion recovery), ST (slice thickness), TSE (turbo spin-echo), DSC (dynamic susceptibility contrast), SE (spin-echo), SPIR (Spectral Presaturation with Inversion Recovery), SV (single-voxel); MV (multivoxel), PRESS (Point Resolved Spectroscopy); FA (flip-angle); WM: white matter; L–W–H: length–width–height.

## References

[1] Weller M., van den Bent M., Preusser M., Le Rhun E., Tonn J.C., Minniti G., Bendszus M., Balana C., Chinot O., Dirven L. (2021). EANO Guidelines on the Diagnosis and Treatment of Diffuse Gliomas of Adulthood. Nat. Rev. Clin. Oncol..

[2] WHO Classification of Tumours Editorial Board (2022). Central Nervous System Tumours: Who Classification of Tumours.

[3] McNamara C., Mankad K., Thust S., Dixon L., Limback-Stanic C., D’Arco F., Jacques T.S., Löbel U. (2022). 2021 WHO Classification of Tumours of the Central Nervous System: A Review for the Neuroradiologist. Neuroradiology.

[4] Ramos A., Hilario A., Lagares A., Salvador E., Perez-Nuñez A., Sepulveda J. (2013). Brainstem Gliomas. Semin. Ultrasound CT MR.

[5] Purohit B., Kamli A.A., Kollias S.S. (2015). Imaging of Adult Brainstem Gliomas. Eur. J. Radiol..

[6] Guillamo J.S., Monjour A., Taillandier L., Devaux B., Varlet P., Haie-Meder C., Defer G.L., Maison P., Mazeron J.J., Cornu P. (2001). Brainstem Gliomas in Adults: Prognostic Factors and Classification. Brain.

[7] Reyes-Botero G., Mokhtari K., Martin-Duverneuil N., Delattre J.-Y., Laigle-Donadey F. (2012). Adult Brainstem Gliomas. Oncologist.

[8] Arvinda H.R., Kesavadas C., Sarma P.S., Thomas B., Radhakrishnan V.V., Gupta A.K., Kapilamoorthy T.R., Nair S. (2009). Glioma Grading: Sensitivity, Specificity, Positive and Negative Predictive Values of Diffusion and Perfusion Imaging. J. Neurooncol..

[9] Chen H.J., Panigrahy A., Dhall G., Finlay J.L., Nelson M.D., Blüml S. (2010). Apparent Diffusion and Fractional Anisotropy of Diffuse Intrinsic Brain Stem Gliomas. AJNR Am. J. Neuroradiol..

[10] Helton K.J., Phillips N.S., Khan R.B., Boop F.A., Sanford R.A., Zou P., Li C.S., Langston J.W., Ogg R.J. (2006). Diffusion Tensor Imaging of Tract Involvement in Children with Pontine Tumors. AJNR Am. J. Neuroradiol..

[11] Damodharan S., Lara-Velazquez M., Williamsen B.C., Helgager J., Dey M. (2022). Diffuse Intrinsic Pontine Glioma: Molecular Landscape, Evolving Treatment Strategies and Emerging Clinical Trials. J. Pers. Med..

[12] Xiao X., Kong L., Pan C., Zhang P., Chen X., Sun T., Wang M., Qiao H., Wu Z., Zhang J. (2021). The Role of Diffusion Tensor Imaging and Tractography in the Surgical Management of Brainstem Gliomas. Neurosurg. Focus.

[13] Hakyemez B., Erdogan C., Ercan I., Ergin N., Uysal S., Atahan S. (2005). High-Grade and Low-Grade Gliomas: Differentiation by Using Perfusion MR Imaging. Clin. Radiol..

[14] Tzika A.A., Aria Tzika A., Astrakas L.G., Zarifi M.K., Zurakowski D., Poussaint T.Y., Goumnerova L., Tarbell N.J., Black P.M. (2004). Spectroscopic and Perfusion Magnetic Resonance Imaging Predictors of Progression in Pediatric Brain Tumors. Cancer.

[15] Porto L., Hattingen E., Pilatus U., Kieslich M., Yan B., Schwabe D., Zanella F.E., Lanfermann H. (2007). Proton Magnetic Resonance Spectroscopy in Childhood Brainstem Lesions. Childs Nerv. System.

[16] Yamasaki F., Kurisu K., Kajiwara Y., Watanabe Y., Takayasu T., Akiyama Y., Saito T., Hanaya R., Sugiyama K. (2011). Magnetic Resonance Spectroscopic Detection of Lactate Is Predictive of a Poor Prognosis in Patients with Diffuse Intrinsic Pontine Glioma. Neuro Oncol..

[17] Piette C., Munaut C., Foidart J.-M., Deprez M. (2006). Treating Gliomas with Glucocorticoids: From Bedside to Bench. Acta Neuropathol..

[18] Kesari S., Kim R.S., Markos V., Drappatz J., Wen P.Y., Pruitt A.A. (2008). Prognostic Factors in Adult Brainstem Gliomas: A Multicenter, Retrospective Analysis of 101 Cases. J. Neurooncol..

[19] Broniscer A., Iacono L., Chintagumpala M., Fouladi M., Wallace D., Bowers D.C., Stewart C., Krasin M.J., Gajjar A. (2005). Role of Temozolomide after Radiotherapy for Newly Diagnosed Diffuse Brainstem Glioma in Children: Results of a Multiinstitutional Study (SJHG-98). Cancer.

[20] Raza S., Donach M. (2009). Bevacizumab in Adult Malignant Brainstem Gliomas. J. Neurooncol..

[21] Stupp R., Mason W.P., van den Bent M.J., Weller M., Fisher B., Taphoorn M.J.B., Belanger K., Brandes A.A., Marosi C., Bogdahn U. (2005). Radiotherapy plus Concomitant and Adjuvant Temozolomide for Glioblastoma. N. Engl. J. Med..

[22] Donaldson S.S., Laningham F., Fisher P.G. (2006). Advances toward an Understanding of Brainstem Gliomas. J. Clin. Oncol..

[23] Laprie A., Pirzkall A., Haas-Kogan D.A., Cha S., Banerjee A., Le T.P., Lu Y., Nelson S., McKnight T.R. (2005). Longitudinal Multivoxel MR Spectroscopy Study of Pediatric Diffuse Brainstem Gliomas Treated with Radiotherapy. Int. J. Radiat. Oncol. Biol. Phys..

[24] Helton K.J., Weeks J.K., Phillips N.S., Zou P., Kun L.E., Khan R.B., Gajjar A., Fouladi M., Broniscer A., Boop F. (2008). Diffusion Tensor Imaging of Brainstem Tumors: Axonal Degeneration of Motor and Sensory Tracts. J. Neurosurg. Pediatr..

[25] Laigle-Donadey F., Doz F., Delattre J.-Y. (2008). Brainstem Gliomas in Children and Adults. Curr. Opin. Oncol..

[26] Mursch K., Halatsch M.-E., Markakis E., Behnke-Mursch J. (2005). Intrinsic Brainstem Tumours in Adults: Results of Microneurosurgical Treatment of 16 Consecutive Patients. Br. J. Neurosurg..

[27] Broniscer A., Gajjar A., Bhargava R., Langston J.W., Heideman R., Jones D., Kun L.E., Taylor J. (1997). Brain Stem Involvement in Children with Neurofibromatosis Type 1: Role of Magnetic Resonance Imaging and Spectroscopy in the Distinction from Diffuse Pontine Glioma. Neurosurgery.

[28] Guillamo J.-S., Créange A., Kalifa C., Grill J., Rodriguez D., Doz F., Barbarot S., Zerah M., Sanson M., Bastuji-Garin S. (2003). Prognostic Factors of CNS Tumours in Neurofibromatosis 1 (NF1): A Retrospective Study of 104 Patients. Brain.

